# Association between gaming disorder and regional homogeneity in highly involved male adult gamers: A pilot resting‐state fMRI study

**DOI:** 10.1002/brb3.3315

**Published:** 2023-11-06

**Authors:** Wen‐tao Jiang, Xia Liu, Zi‐yun Xu, Zhi‐feng Zhou, Chang‐jun Tie, Xiao‐ying Liu, Ji‐hui Yang, Hai Li, Wen‐tao Lai

**Affiliations:** ^1^ Department of Radiology Shenzhen Mental Health Center/Shenzhen Kangning Hospital Shenzhen Guangdong China; ^2^ Institute of Computing Technology Chinese Academy of Sciences Beijing China; ^3^ Peng Cheng Laboratory Shenzhen Guangdong China; ^4^ Department of Drug Dependence Shenzhen Mental Health Center/Shenzhen Kangning Hospital Shenzhen Guangdong China; ^5^ Beijing Intelligent Brain Cloud, Inc. Beijing China

**Keywords:** depression, gaming disorder, highly involved gamers, hippocampus, impulsivity, regional homogeneity

## Abstract

**Background:**

Gaming behavior can induce cerebral changes that may be related to the neurobiological features of gaming disorder (GD). Additionally, individuals with higher levels of depression or impulsivity are more likely to experience GD. Therefore, the present pilot study explored potential neurobiological correlates of GD in the context of depression and impulsivity, after accounting for video gaming behavior.

**Methods:**

Using resting‐state functional magnetic resonance imaging (fMRI), a cross‐sectional study was conducted with 35 highly involved male adult gamers to examine potential associations between GD severity and regional homogeneity (ReHo) in the entire brain. A mediation model was used to test the role of ReHo in the possible links between depression/impulsivity and GD severity.

**Results:**

Individuals with greater GD severity showed increased ReHo in the right Heschl's gyrus and decreased ReHo in the right hippocampus (rHip). Furthermore, depression and impulsivity were negatively correlated with ReHo in the rHip, respectively. More importantly, ReHo in the rHip was found to mediate the associations between depression/impulsivity and GD.

**Conclusions:**

These preliminary findings suggest that GD severity is related to ReHo in brain regions associated with learning/memory/mood and auditory function. Higher levels of depression or impulsivity may potentiate GD through the functional activity of the hippocampus. Our findings advance our understanding of the neurobiological differences behind GD symptoms in highly involved gamers.

## Graphical abstract

35 highly involved male adult gamers participated in this pilot study. Hippocampal dysfunction related to the gaming disorder severity, depression and impulsivity. Hippocampal dysfunction may play a critical role in the development of gaming disorder.

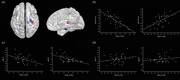

## 1 INTRODUCTION

According to the 11th edition of the International Classification of Diseases (ICD‐11), gaming disorder (GD) is characterized by the loss of control over gaming, the precedence of gaming over other daily activities, and the continuation/escalation of gaming despite negative consequences (World Health Organization, 2019). Several studies have suggested that GD is associated with changes in the brain regions responsible for response‐inhibition, emotion regulation, cognitive control, working memory, decision‐making, visual and auditory functions, and the reward system (Kuss et al., 2018; Qin et al., 2020; von Deneen et al., 2022; Weinstein & Lejoyeux, 2020; Weinstein et al., 2017; Yan et al., 2021; Yao et al., 2017; Zheng et al., 2019). Moreover, theoretical models, such as the Interaction of person–affect–cognition–execution (I‐PACE) model of GD, have been proposed to describe the psychological and neurobiological processes underlying the development and maintenance of GD (Brand et al., 2019; Brand et al., 2016).

These findings have provided insights into the biological mechanisms underlying GD. However, the findings are highly heterogeneous across individuals with GD, in part due to such issues as (1) the GD diagnostic criteria from the fifth edition of the Diagnostic and Statistical Manual of Mental Disorders (DSM‐5) (American Psychiatric Association, 2013) may not sufficiently distinguish between high and problematic involvement in video gaming, which could lead to the pathologization of non‐problematic patterns of gaming (Billieux et al., 2019; Castro‐Calvo et al., 2021; Deleuze et al., 2018). Furthermore, a multicenter cohort study reported that 32.4% of participants met the GD criteria of the DSM‐5, whereas only 6.4% met the more stringent GD criteria of the ICD‐11 (Jo et al., 2019). This suggests that the GD group, usually defined by the DSM‐5 and/or screening tools, may include many highly involved gamers rather than individuals with GD. (2) Playing video games could induce alterations in multiple brain regions that are similar to the brain regions associated with GD, mainly located in the prefrontal cortex, striatum, and limbic system (Brilliant et al., 2019; Kovbasiuk et al., 2022; Palaus et al., 2017). Compared with non‐gamers, extensive gamers (those who play about 3 h/day) presented alterations of brain structure in the prefrontal cortex (He et al., 2021). A combined cross‐sectional and longitudinal study found that regular gamers (RGs) show hyperactive function within a parietal network, including the posterior cingulate cortex, in response to game stimuli (Yu et al., 2021). Furthermore, game‐playing time has been demonstrated to be significantly associated with specific brain structures (Kühn & Gallinat, 2014; Lee et al., 2018; Takeuchi et al., 2016) and functions (Meng et al., 2015; Shin et al., 2021). Therefore, comparing GDs (based on the DSM‐5 criteria) with controls (in particular, gaming‐naive controls), may confuse the neurobiological characteristics of game‐playing behavior with those of GD.

Regional homogeneity (ReHo) represents the temporal homogeneity of the regional blood oxygen level–dependent signal detected by functional magnetic resonance imaging (fMRI); it may reflect neural activity during the paradigm‐free resting state (Zang et al., 2004). ReHo has been successfully used to investigate normal brain functioning and to assess dysfunction in neurological and psychiatric disorders, such as major depressive disorder (Geng et al., 2019), schizophrenia (Huang et al., 2022), and substance use disorder (Sanvicente‐Vieira et al., 2022). Earlier research has found that GD individuals exhibit differences in ReHo in widespread brain regions compared to healthy controls or RGs, which appear, by comparison, to be highly heterogeneous (Dong et al., 2012; Kim et al., 2015; Liu et al., 2010; Wang et al., 2019). One recent study has demonstrated that ReHo‐based multi‐voxel pattern analysis (MVPA) can distinguish GD subjects from RGs, and that the ReHo values of the regions that contributed most to the classification, including the bilateral parahippocampal gyrus (PG), right anterior cingulate cortex, middle frontal gyrus (MFG), and left cerebellum posterior lobe, were significantly associated with addiction severity in all participants (Wang et al., 2020). Meanwhile, another study found that, compared to the use of DSM‐5 scores ≥5 as the inclusion criteria for GD subjects, DSM‐5 scores ≥6 could enhance the ReHo‐based MVPA classification accuracy (Dong et al., 2020). Most notably, the cerebral regions that contributed the most to classifications in these two ReHo‐based MVPA studies were the MFG and PG. The former is related to impulsivity and strategic behaviors (Steinbeis et al., 2012), whereas the latter plays a critical role in representing episodic memories and using those memories for future adaptive behavior (Lee et al., 2021). These findings suggest that more stringent criteria for GD (from screening tools to DSM‐5) and control of gaming behavior (from game‐naïve controls to RGs) may reduce heterogeneity and approximate the neurobiological correlates of GD (from widespread brain regions to several critical regions) (see Table 1).

**TABLE 1:** Previous regional homogeneity (ReHo) studies in patients with gaming disorder (GD).

Study	Clinical, *n*(m:f), age (mean ± SD), game‐playing time (mean ± SD)	Control, *n*(m:f), age (mean ± SD), game‐playing time (mean ± SD)	Diagnostic criteria	Findings
Liu et al. (2010)	GD, 19 (11:8), 21.0 ± 1.3, NS	Sex‐matched subjects, 19 (11:8), 20.0 ± 1.3, NS	Modified YDQ	Enhanced ReHo in the cerebellum, brainstem, right cingulate gyrus, bilateral parahippocampus, right frontal lobe (rectal gyrus, inferior frontal gyrus and middle frontal gyrus), left superior frontal gyrus, left precuneus, right postcentral gyrus, right middle occipital gyrus, right inferior temporal gyrus, left superior temporal gyrus, and middle temporal gyrus
Dong et al. (2012)	GD, 15 (15:0), 24.2 ± 3.5, NS	Controls scored lower than 20 in IAT, 14 (14:0), 24.6 ± 3.8, NS	IAT	Enhanced ReHo in the brainstem, inferior parietal lobule, left posterior cerebellum, and left middle frontal gyrus Decreased ReHo in the temporal, occipital, and parietal cortices
Kim et al. (2015)	GD, 16 (16:0), 21.63 ± 5.92, 45.95 ± 15.87 h/week	Healthy controls, 15 (15:0), 25.40 ± 5.29, less than 2 h/day	DSM‐5 + IAT + game‐playing time	Enhanced ReHo in the left posterior cingulate cortex Decreased ReHo in the right superior temporal gyrus
Wang et al. (2019)	GD, 46 (23:23), male 23.0 ± 1.14, female 22.7 ± 1.41, male 22.5 ± 7.59 h/week, female 16.9 ± 5.06 h/week	RGs, 58 (29:29), male 23.4 ± 1.47, female 22.6 ± 1.68, male 18.1 ± 7.50 h/week, female 15.7 ± 4.71 h/week	DSM‐5 + IAT + game‐playing time	male GD: Enhanced ReHo in the left middle occipital gyrus and right middle temporal gyrus. Decreased ReHo in the right posterior cingulate cortex female GD: Decreased ReHo in the left middle occipital gyrus and right middle temporal gyrus
Wang et al. (2020)	GD, 103 (57:46), 21.06 ± 2.35, 19.33 ± 9.40 h/week	RGs, 99 (51:48), 21.45 ± 2.31, 20.70 ± 9.57 h/week	DSM‐5 + IAT + game‐playing time	Enhanced ReHo in the right anterior cingulate cortex and right middle frontal gyrus Decreased ReHo in the bilateral parahippocampal gyrus and left cerebellum posterior lobe
Dong et al. (2020)	GD, 148 (86:62), 21.25 ± 2.455, 8.264 ± 3.641 h/week	RGs, 226 (141:85), 21.60 ± 2.484, 6.146 ± 3.063 h/week	DSM‐5 + IAT + game‐playing time	The most informative regions for classification between GD subjects and RGs included the following: bilateral inferior cerebellum, superior frontal gyrus, cuneus, inferior temporal gyrus, middle frontal gyrus and parahippocampal gyrus

Abbreviations: DSM‐5, Diagnostic and Statistical Manual of Mental Disorders, fifth‐edition; IAT, Internet Addiction Test; NS, not stated; RGs, regular gamers; YDQ, Young Diagnostic Questionnaire.

Depression and impulsivity, as vulnerability factors for GD, have been found to be highly correlated with GD symptoms (Gervasi et al., 2017; González‐Bueso et al., 2018; Jeong et al., 2021; Jo et al., 2019; Ostinelli et al., 2021; Petry et al., 2018; Wu et al., 2018). More importantly, higher levels of depression or impulsivity may increase the risk of developing GD (Jeong et al., 2021; Paulus et al., 2018; Ryu et al., 2018). Interestingly, strong associations between depression and impulsivity also have been observed in the healthy population (Granö et al., 2007; Moustafa et al., 2017) and in depressed individuals (Saddichha & Schuetz, 2014; Swann et al., 2008). Meta‐analytic evidence indicates that significant depression‐related changes in ReHo can be found within the medial prefrontal cortex (Iwabuchi et al., 2015), orbitofrontal cortex, and hippocampus (Gray et al., 2020; Hao et al., 2019). In addition, impulsivity was found to be related to alterations of prefrontal function in GD (Ding et al., 2014) and in drug use disorder (Büchel et al., 2017; Du et al., 2022). Taken together, these findings indicate that depression and impulsivity are associated with ReHo alterations in brain regions that are similar to those that exhibit ReHo changes in GD, including the hippocampus and prefrontal cortex. This points to brain functional alterations as a potential adverse consequence of depression or impulsivity that might contribute to vulnerability to GD. It indicates that risk factors, such as depression and impulsivity, may play critical roles in the neural mechanism underlying the thus far poorly understood development of GD.

To address these issues, this pilot study aimed to examine the association between ReHo and the severity of GD in highly involved male adult gamers. We were interested in exploring the underlying neurobiological mechanism of GD after controlling the influence of game‐playing behavior. We hypothesized that (1) GD severity is correlated with alterations in ReHo in the prefrontal cortex and hippocampus; (2) these alterations are associated with depression and impulsivity; (3) depression/impulsivity influences GD severity through the prefrontal and hippocampal activity measured by ReHo.

## 2 MATERIALS AND METHODS

### 2.1 Participants

**FIGURE 1:**
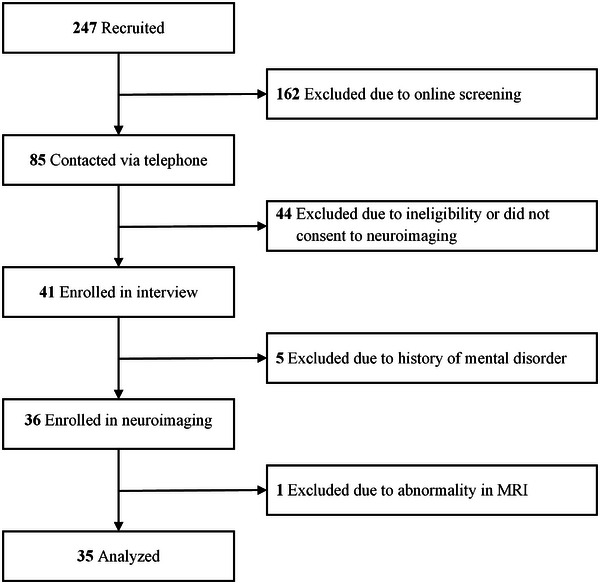
Flowchart of participants included in analysis of brain function.

The procedures of this study were carried out in accordance with the Declaration of Helsinki. The protocol of this study was approved by the Human Ethics Committee of Shenzhen Kangning Hospital (No. 2020‐K031‐01), and written informed consent was obtained from all participants. Two hundred and forty‐seven participants were recruited through posters, online advertisements, and outpatients of addiction medicine at Shenzhen Kangning Hospital. First, 85 male gamers passed the preliminary online screening and were contacted via telephone to determine whether they met the following criteria: (1) age between 18 and 36 years, which is representative of the adult population with GD (Wang & Cheng, 2021); (2) game experience (playing games ≥14 h/week for ≥1 year); (3) mainly playing popular games (such as League of Legends or Arena of Valor) but not logic/puzzle games or tabletop games. Then, 41 gamers agreed to participate and underwent a Mini‐International Neuropsychiatric Interview (Sheehan et al., 1998) to exclude those with a prior history of mental disorders (*n* = 5). Furthermore, one subject was excluded from the study due to an abnormality in his MRI. Additional exclusion criteria for all participants included contraindications to MRI scanning, neurological disorders, cardiovascular disease, and diabetes. Ultimately, 35 eligible participants were included in the MRI analysis (exclusion of participants detailed in Figure 1).

### 2.2 Clinical assessments

In the present study, the Chinese version of the Internet Gaming Disorder Test (IGD‐20 Test) (Pontes et al., 2014) was administered to measure the severity of GD. The IGD‐20 Test includes 20 items incorporating the theoretical framework of the components model of addiction (i.e., salience, mood modification, tolerance, withdrawal symptoms, conflict, and relapse) (Griffiths, 2005). The Barratt Impulsiveness Scale version 11 (BIS‐11) (Patton et al., 1995) and the Beck Depression Inventory version II (BDI‐II) (Beck et al., 1996) were administered to assess the trait of impulsivity and depression of all participants, respectively. Handedness was ascertained by the Edinburgh Handedness Inventory—short form. All assessments were administered on the same day as the MRI scan.

### 2.3 MRI data acquisition

MRI scanning was conducted on a 3.0‐Tesla Discovery MR750 system (General Electrical Healthcare) equipped with an eight‐channel phased‐array head coil at the Neuropsychiatry Imaging Center of Shenzhen Kangning Hospital. During the resting‐state fMRI scanning, participants were asked to keep their eyes open and to relax without falling asleep or thinking about specific things. The functional images were acquired with the following parameters: repetition time (TR) = 2000 ms, echo time (TE) = 25 ms, flip angle (FA) = 90°, field of view (FOV) = 220 × 220 mm^2^, matrix = 64 × 64, slice thickness/gap = 3.2/0 mm, number of slices = 48, 240 volumes (8 min). A high‐resolution T1‐weighted structural scan was acquired for co‐registration purposes using a three‐dimensional fast spoiled gradient‐recalled sequence (192 slices, TR = 6.7 ms, TE = 2.9 ms, FA = 12°, FOV = 256 × 256 mm^2^, voxel size = 1 mm × 1 mm × 1 mm resolution).

### 2.4 Imaging data preprocessing

Data preprocessing was performed using the Configurable Pipeline for Analysis of Connectomes (htttps://fcp‐indi.github.com), a Python‐based pipeline tool comprising AFNI (Cox, 1996), ANTs (Tustison et al., 2014), FSL (Jenkinson et al., 2012), and custom Python code. The whole analysis was accelerated and simplified using the NeuroScholar cloud platform (http://www.neuroscholar.cn, Beijing Intelligent Brain Cloud, Inc.).

Specifically, functional preprocessing comprised the following steps: (1) The first 10 volumes were removed, and slice timing correction was performed. (2) Motion correction was performed on averaged images to obtain motion parameters. The data of all participants satisfied the criteria of less than 2 mm translational movement and 2° rotational movement. (3) Skull stripping was performed, and global mean intensity was normalized to 10,000. (4) Functional images were registered to anatomical space using linear transformation, white matter (WM) boundary‐based transformation, and the prior WM tissue segmentation from FSL, normalized to the Montreal Neurological Institute (MNI) stereotactic space, and then resampled to a voxel size of 3 × 3 × 3 mm^3^. (5) Motion artifacts were removed using the ICA‐based strategy for Automatic Removal of Motion Artifacts with partial component regression (Pruim et al., 2015). (6) Nuisance signal regression was applied, including (a) mean values from the signal in WM and in cerebrospinal fluid, (b) 24 motion parameters, (c) linear trends in the time series, and (d) global signal only for one set of strategies. (7) Band‐pass temporal filtering between 0.01 and 0.08 Hz was applied.

### 2.5 Analysis of ReHo

ReHo measures the degree of coherence of local regional neural activity (Zang et al., 2004), defined as Kendall's coefficient of concordance of the time series of a given voxel to those of its nearest 26 neighbor voxels. For purposes of standardization, the ReHo value of each voxel was converted into a *Z*‐score. Finally, the resulting ReHo images were spatially smoothed with a 6 mm full width at half‐maximum Gaussian kernel.

### 2.6 Statistical analysis

Given that our primary objective was to examine the association between GD severity and ReHo in the brain, the IGD‐20 Test score was used as a participant‐level regressor, and age and joystick‐year were added as noninterest covariates in a multiple regression model in Statistical Parametric Mapping software (SPM12; http://www.fil.ion.ucl.ac.uk/spm). Joystick‐year, used as an estimate of the dosage of video gaming over the lifetime, was defined as the square root of the outcome of hours of game playing per week × 52 (weeks per year) × years (Kühn & Gallinat, 2014). To account for the robustness, this analysis was repeated with no covariates. The resultant statistical map was set at a whole‐brain uncorrected voxel threshold of *p* < .001 with at least 20 contiguous voxels per cluster.

Partial correlations were calculated to explore associations between extracted mean ReHo values from significant clusters in the above analysis and the scores on the IGD‐20 Test, BIS‐11, and BDI‐II. A mediation analysis was finally conducted to investigate the relationships among GD, depression/impulsivity, and the related neural bases. The significance of the mediation effect was evaluated by the PROCESS macro (Hayes, 2013), with bootstrapping (5000 resamples) and bias‐corrected 95% confidence intervals (CIs). All partial correlations and mediation analyses were controlled for age and joystick‐year. A significant difference was defined as a two‐tailed *p* value <.05, and the analyses were conducted in SPSS, version 26 (IBM Corp).

## 3 RESULTS

### 3.1 General data

Descriptive statistics of the entire sample are shown in Table 2. Participants reported a mean (standard deviation [SD]) impulsivity of 71.40 (17.24) and showed levels of current depression (mean [SD] BDI‐II, 5.46 [5.87]). Moreover, the association between impulsivity and depression was significant (*r* = .72; *p* < .001). IGD‐20 Test scores ranged from 20 to 86 and were significantly correlated with body mass index (BMI: *r* = .41; *p* = .02), impulsivity (BIS‐11: *r* = .60; *p* < .001), and depression (BDI‐II: *r* = .55; *p* = .001), but not with age (*r* = −.17; *p* = .34), education (*r* = −.03; *p* = .85), or joystick‐year (*r* = −.06; *p* = .74).

**TABLE 2:** Demographic and clinical characteristics.

Characteristic	Value (*N* = 35)
Age, years, mean (SD), [range]	23.77 (4.17) [19–33]
Education, years, mean (SD), [range]	15.26 (1.27) [12–19]
BMI, kg/m^2^, mean (SD), [range]	24.02 (3.81) [18.32–34.57]
Smoking, yes/no	6/29
Drinking, yes/no	2/33
Handedness, right/left/mixed	32/0/3
Game playing per week, hours, mean (SD), [range]	26.23 (10.78) [14–56]
Gaming history, months, mean (SD), [range]	66.83 (55.03) [12–288]
Joystick‐year^a^, mean (SD), [range]	80.61 (34.04) [27.93–161.89]
IGD‐20 Test, mean (SD), [range]	54.34 (18.28) [20–86]
BIS‐11, mean (SD), [range]	71.40 (17.24) [42–118]
BDI‐II, mean (SD), [range]	5.46 (5.87) [0–22]

Abbreviations: BDI‐II, Beck Depression Inventory version II; BIS‐11, Barratt Impulsiveness Scale version 11; BMI, body mass index; IGD‐20 Test, Internet Gaming Disorder Test; SD, standard deviation.

^a^ $\mathrm{Joystick}-\mathrm{year}=\sqrt{\mathrm{Game}\;\mathrm{playing}\;\mathrm{per}\;\mathrm{week}\;\mathrm{hours}\;\times \;52\;\times \;\mathrm{years}}\;$.

### 3.2 Associations between ReHo and GD severity

**FIGURE 2:**
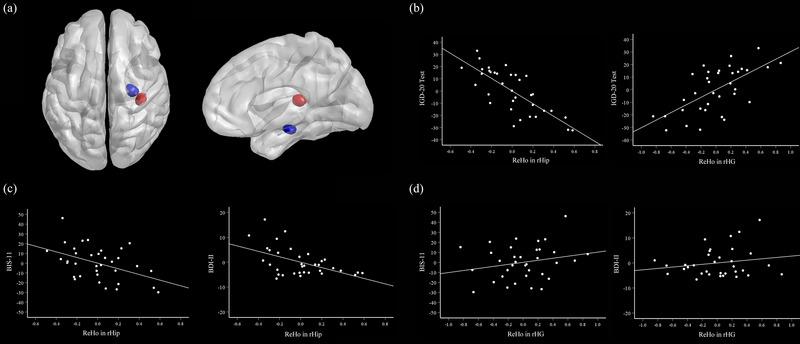
Associations between regional homogeneity (ReHo) of resting‐state brain activity with gaming disorder (GD) severity and depression/impulsivity. (a) Whole‐brain correlations between ReHo and GD severity. Positive correlation region within the right Heschl's gyrus (rHG) in red and negative correlation region within the right hippocampus (rHip) in blue. (b) ReHo in the rHip negatively correlated with Internet Gaming Disorder Test (IGD‐20 Test) (*r* = −.77, *p* < .001), whereas ReHo in the rHG positively correlated with IGD‐20 Test (*r* = .64, *p* < .001), seen in the partial correlation plot of residuals. (c) ReHo in the rHip negatively correlated with Barratt Impulsiveness Scale version 11 (BIS‐11) (*r* = −.45, *p* = .008) and Beck Depression Inventory version II (BDI‐II) (*r* = −.51, *p* = .002), seen in the partial correlation plot of residuals. (d) Scatter plots of ReHo in the rHG and BIS‐11 (*r* = .23, *p* = .20), BDI‐II (*r* = .19, *p* = .30), with residuals.

After accounting for age and joystick‐year, greater GD severity was significantly associated with increased ReHo within the right Heschl's gyrus (rHG) and reduced ReHo within the right hippocampus (rHip) (see Table 3). Figure 2a illustrates the ReHo in the brain regions that showed significant correlations with IGD‐20 Test scores. Figure 2b illustrates the significant correlations between IGD‐20 Test scores and ReHo in the rHG (*r* = .64, *p* < .001) and in the rHip (*r* = −.77, *p* < .001). Moreover, additional analysis was performed to examine the GD‐related ReHo map without controlling age and joystick‐year. These findings were sustained when the analysis was repeated with no covariates (Table 4).

**TABLE 3:** Anatomical locations of voxel‐wise association between regional homogeneity (ReHo) and gaming disorder (GD) severity (age and joystick‐year covaried).

Characteristic	Brodmann area	MNI coordinate			*p* Value	Cluster size (mm^3^)
		*x*	*y*	*z*		
Positive correlation						
Right Heschl's gyrus	41	39	−24	12	*p _uncorrected_* < 0.001	945
Negative correlation						
Right hippocampus	NA	27	−15	−18	*p _FWE_* = 0.016	1701

*Note*: *p_uncorrected_*: uncorrected; *p_FWE_*: cluster level family wise error corrected <.05.

Abbreviations: MNI, Montreal Neurological Institute; NA, not applicable.

**TABLE 4:** Anatomical locations of voxel‐wise association between regional homogeneity (ReHo) and gaming disorder (GD) severity (no covariates).

Characteristic	Brodmann area	MNI coordinate			*p* Value	Cluster size (mm^3^)
		*x*	*y*	*z*		
Positive correlation						
Right Heschl's gyrus	41	39	−27	12	*p_uncorrected_* < .001	999
Negative correlation						
Left superior frontal gyrus	6	−18	30	63	*p_uncorrected_* < .001	594
Right hippocampus	NA	27	−15	−18	*p_FWE_* = .016	1728

*Note*: *p_uncorrected_*: uncorrected; *p_FWE_*: cluster level family wise error corrected <.05.

Abbreviations: MNI, Montreal Neurological Institute; NA, not applicable.

### 3.3 Associations between ReHo and depression/impulsivity

In order to determine whether the ReHo results are based on the associated impulsivity and depression, the extracted mean ReHo values from the clusters were correlated with BIS‐11 and BDI‐II scores after accounting for age and joystick‐year. ReHo in the rHip was negatively correlated with BIS‐11 scores (*r* = −.45, *p* = .008) and BDI‐II scores (*r* = −.51, *p* = .002). No significant correlations were observed between ReHo in the rHG and either BIS‐11 scores (*r* = .23, *p* = .20) or BDI‐II scores (*r* = .19, *p* = .30). The partial correlation plots with residuals of the above results are shown in Figure 2c,d.

### 3.4 Mediation analysis

**FIGURE 3:**
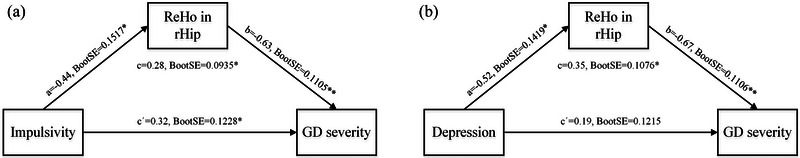
Standardized coefficients and bootstrap standard errors for each path of the two mediation models. Path c represents the indirect effect and path c′ represents the direct effect. (a) Mediation effect of regional homogeneity (ReHo) in the right hippocampus (rHip) on the association between impulsivity and gaming disorder (GD) severity. (b) Mediation effect of ReHo in the rHip on the association between depression and GD severity. BootSE, bootstrap standard errors. *p < .05, **p < .001.

Based on our hypotheses and the above findings, a mediation analysis was conducted to determine whether the ReHo in the rHip mediated the relationships between impulsivity/depression and GD severity. Our results showed that when impulsivity was examined as a predictor, the ReHo in the rHip significantly mediated the relationship between impulsivity and GD (indirect effect ratio: 46.67%, CI [.0804, .4581]; direct effect ratio: 53.33%, CI [.0951, .5774]; Figure 3a). In addition, when depression was examined as a predictor, the mediating effect of ReHo in the rHip in the link between depression and GD was significant (indirect effect ratio: 64.16%, CI [.1670, .5945]; direct effect ratio: 35.84%, CI [−.0748, .4215]; see Figure 3b).

## 4 DISCUSSION

To the best of our knowledge, this is the first study to examine associations of GD severity with whole‐brain ReHo patterns in an adult population with high involvement in video gaming, and to provide an empirically based concept that links the risk factors and brain functional alterations with GD severity. We preliminarily observed that gamers who reported more GD severity showed decreased ReHo in the rHip, a brain area that is essential for learning/memory/mood, and increased ReHo in the rHG, which plays a critical role in auditory function, even after accounting for variance associated with age and game‐playing time. Furthermore, ReHo in the rHip was negatively associated with depression and impulsivity and could mediate the prospective relationships between depression/impulsivity and GD. Our study improves our understanding of the GD‐related changes in highly involved gamers and sheds light on potential mechanisms underlying the associations between depression/impulsivity and GD.

The finding of an association between GD severity and ReHo in the rHip was in‐line with recent studies that used ReHo‐based MVPA to distinguish GD subjects from RGs based on differences in the right PG (Dong et al., 2020; Wang et al., 2020). One of these studies also found ReHo in the right PG to be negatively associated with addiction severity in all participants (Wang et al., 2020). The hippocampus is a vital brain structure that sends projections to both the cortex and striatum to coordinate learning, memory, and mood. Accumulated evidence supports the involvement of the hippocampus in the development and maintenance of substance use disorder (Avchalumov & Mandyam, 2021; Dai et al., 2022; Peyton et al., 2021). Additionally, the reactivation of withdrawal memory is considered one of the most important reasons for drug relapse, and the hippocampus might participate in memory reactivation, which plays a potential role in shifting motivational processing from seeking positive reinforcement to avoiding the aversive effects of withdrawal (Dai et al., 2022; Kutlu & Gould, 2016; Pantazis et al., 2021). As such, our finding highlights the hippocampus‐dependent learning deficit in highly involved gamers with greater GD severity.

A positive association between ReHo value and GD severity was identified in the rHG, a convolution on the superior temporal plane that hosts the primary auditory cortex. More importantly, the auditory cortex, including Heschl's gyrus and the superior temporal gyrus (STG), plays a key role in the integration of auditory and visual cues and in the perception of emotion based on audiovisual information (Gao et al., 2019; Mauchand & Zhang, 2022). There is evidence that alteration of ReHo in the STG is a candidate neurobiological marker for GD, differentiating individuals with this disorder from those with alcohol use disorder and healthy controls (Kim et al., 2015). Moreover, GD individuals exhibit hyperactivation in the insula, STG, and superior parietal lobe while watching game‐related videos (Liu et al., 2016). As no significant correlations were observed between ReHo in the rHG and depression or impulsivity, we prefer an explanation based on a compensatory mechanism of the brain to balance dysfunction within the rHG in GD rather than an increased emotional involvement and processing related to abnormal auditory in the current study (Oertel‐Knöchel et al., 2014). Thus, the alteration of ReHo in the rHG may be related to impairments in higher audiovisual information processing in gamers with GD.

A point to be considered is that, unlike prior studies that reported alterations of functional activity in the prefrontal cortex in GD (Dong et al., 2020; Kuss et al., 2018; Wang et al., 2020; Weinstein & Lejoyeux, 2020; Weinstein et al., 2017; Yao et al., 2017), our study did not find any associations between ReHo in the prefrontal cortex and GD, although the ReHo in the left superior frontal gyrus was found in the analysis with no covariates. One possible explanation for this disagreement is that, in the updated I‐PACE model, earlier research on GD was based on the definition in the DSM‐5. That definition may correspond to the early stages of GD, which are involved in general inhibitory control (as mediated by the prefrontal cortex), whereas later stages of GD are related to diminished specific stimulus‐related inhibitory control instead of general inhibitory control (Brand et al., 2019). Notably, ReHo is based on the resting state (no stimuli and no tasks), but not on game‐related paradigms. Nevertheless, a more ideal approach would be a case–control study between GD individuals, as defined by the ICD‐11, and highly involved gamers in a large sample. We expect that the findings of the current study will provide a priori data for such a future study.

Our findings of associations between depression/impulsivity and GD severity were in‐line with multiple prior reports, including meta‐analytic and longitudinal evidence, supporting the hypothesis that depression or impulsivity is related to an increased risk of developing GD (González‐Bueso et al., 2018; Jeong et al., 2021; Ostinelli et al., 2021; Paulus et al., 2018). The results presented here highlight hippocampal abnormality as an adverse consequence of depression, a notion that is supported by meta‐analyses that have shown associations between depression and altered brain function (Gray et al., 2020; Hao et al., 2019). Furthermore, hippocampal dysfunction was found in this study to be a complete mediator of the relationship between depression and GD severity, suggesting that a higher degree of depression is related to dysfunction in the hippocampus, thereby increasing the risk of GD. In addition, ReHo in the rHip was found to be a partial mediator of the relationship between impulsivity and GD severity. This is inconsistent with a prior study reporting a close association between impulsivity and prefrontal function in GD (Ding et al., 2014). A potential explanation is that the hippocampus has intensive connections with the prefrontal cortex, and these two regions participate jointly in the control of executive function, memory, and mood in addiction (Peyton et al., 2021). Another explanation may be the high association between impulsivity and depression in our sample. Although longitudinal studies on depression/impulsivity, brain function, and GD are scarce, the concept proposed here is similar to that explored in several longitudinal studies that have demonstrated the role of brain functional alterations as an intermediate phenotype connecting depression/impulsivity and future development of other addictive behaviors (Qu et al., 2016; Whelan et al., 2014; Worhunsky et al., 2016).

This study has some limitations that must be considered. First, it included only a small number of participants. As this study was a pilot investigation, we intend to proceed with a follow‐up study with a large sample to assess the neurobiological correlates of GD in the future. Second, we did not examine anatomical changes that are also correlated with GD severity. Thus, promising avenues of future investigation include brain structure and other neuroimaging combined with a variety of analyses to explore different aspects of the neural mechanisms underpinning GD. In addition, the current study did not use a broad assessment of game‐playing behavior. A more detailed measurement of game genres and devices is warranted to characterize the specificity of neurobiological changes in response to these factors, as these also may be linked to specific alterations of brain volume and function. Finally, the interpretation of the relationships among the risk factors, ReHo, and GD should be cautious due to the cross‐sectional design. Our study could only provide possible theoretical models of development in GD, but not determine the causality by spontaneous regional fluctuations or the mediation model. In the future, longitudinal studies should be conducted to examine the associations between risk factors (e.g., personality traits, depression, anxiety, familial environment) and GD with neurobiological effects in the development from childhood to adulthood.

## 5 CONCLUSIONS

This pilot study of 35 highly involved male adult gamers found that those who experienced greater GD severity showed decreased ReHo within the rHip and increased ReHo within the rHG, controlling for age and game‐playing time. Furthermore, this study provides preliminary evidence for a clinically relevant relationship among depression/impulsivity, brain function, and GD, showing hippocampal activity as a mediator between depression/impulsivity and GD symptoms. These findings may suggest a potential neural correlate underlying the high risk for GD in highly involved gamers.

## AUTHOR CONTRIBUTIONS

**Wen‐tao Jiang**: Conceptualization; data curation; funding acquisition; methodology; project administration; writing—original draft. **Xia Liu**: Conceptualization; data curation; methodology; project administration; writing—review and editing. **Zi‐yun Xu**: Data curation; formal analysis; visualization. **Zhi‐feng Zhou**: Investigation. **Chang‐jun Tie**: Conceptualization; methodology. **Xiao‐ying Liu**: Investigation. **Ji‐hui Yang**: Investigation. **Hai Li**: software; Data curation; formal analysis. **Wen‐tao Lai**: Conceptualization; funding acquisition; data curation; formal analysis; project administration; supervision; writing—review and editing.

## CONFLICT OF INTEREST STATEMENT

The authors declare no conflicts of interest.

### PEER REVIEW

The peer review history for this article is available at https://publons.com/publon/10.1002/brb3.3315.

## Data Availability

The raw/processed data cannot be shared due to the nature of this research, participants of this study did not agree for their data to be shared publicly and the data also formed part of an ongoing study.
